# 

**Published:** 1899-10

**Authors:** 


### NOTICES.

### Union Meeting Seventh and Eighth District Dental Societies, State of New York.

The thirty-second union meeting of the above societies will be held in the assembly room of the New Osburn House, Rochester, N. Y., Tuesday, Wednesday and Thursday, October 24, 25 and 26, 1899.

PRELIMINARY ANNOUNCEMENT.

1. Cements, Dr. J. H. Beebee, Rochester; 2. Subject to be announced, Dr. J. Wright Beach, Buffalo; 3. Cosmetic Dentistry, Prof. Charles H. Ward, Rochester; 4. Subject to be announced, Dr. W. A. Barrows, Buffalo; 5. Treatment of Fractures of Lower Maxilla, Dr. Frank Greene, Geneva; 6. Subject to be announced, Dr. S. E. McDougall, Buffalo; 7. Anesthetics in Dental Practice, Dr. J. F. Knapp, Geneva; 8. Subject to be announced, Dr. . . Preston, Buffalo; 9. The Use and Limitations of Formaldehyde in Dentistry, Dr. F. W. Low, Buffalo; 10. Subject to be announced, Dr. R. H. Hofheinz, Rochester; 11. The Embryological Development of the Dental Tissues, Dr. W. C. Barrett, Buffalo; 12. Subject to be announced, Dr. Leroy Requa, Rochester; 13. Articulation, Dr. George B. Snow, Buffalo; 14. Subject to be announced, Dr. W. H. Povall, Mt. Morris; 15. Subject to be announced, Dr. C. H. Nicholson, Rochester.

The committee, in addition, have under arrangement other important additions. There will be clinics in abundance, together with a complete dental exhibit. The committee are making strenuous efforts to make this one of the best union meetings ever held by the society, and well worthy of your attendance. Members of the profession are cordially invited.

WM. W. BELCHER, *Chairman*,
827 Granite Building, Rochester, N. Y.

### Examinations.

The Pennsylvania Board of Dental Examiners will conduct examinations simultaneously in Philadelphia and Pittsburg, December 18th, 19th and 20th. Applications for examination must be made to Hon. James W. Latta, Secretary of the Dental Council, Harrisburg, Pa.

G. W. KLUMP, *Sec'y*,
Williamsport, Pa.

Now, it is to be hoped that every member of the profession not a member of the Protective Association will at once become so, by applying to Dr. J. N. Crouse, of Chicago, Chairman of the Dental Protective Association. He will be found, for the present at least, with the gate open for the entrance of all who desire to escape from an unjust prosecution.

### Pennsylvania State Dental Socieiy.

111 N. 5th St., Reading, Pa., October 12, 1899.

Dear Doctor:—The next meeting of the Pennsylvania State Dental Society will be held at the Neversink Mountain Hotel, near this city, July 10, 11 and 12, 1900. For list of officers see current number of *Dental Cosmos*.

Yours, C. V. Kratz, *Rec. Sec'y.*

### Ohio State Dental Society—Annual Meeting.

The thirty-fourth annual meeting of the Ohio State Dental Society will be held at Columbus, Ohio, December 5, 6 and 7, 1899, in the Great Southern Hotel. All are cordially invited.

S. D. Ruggles, *Secretary.*

We will call special attention to the above announcement. The occasion of this meeting will be an interesting and very important one. There are various professional matters that should have special consideration, and we trust the members will not only come prepared with papers, and to carry out the ordinary work of such occasions, but that they will come specially prepared to discuss the educational and legal interests of the profession.

The Dental Protective Association is assuming a very important attitude at the present time, as will be seen by reference to a statement on another page. Let every one come with a pronounced opinion on this subject and with the determination to be a member of the Dental Protective Association, and to do whatever he can for its best interest.

